# Radium-223 Therapy for Patients with Metastatic Castrate-Resistant Prostate Cancer: An Update on Literature with Case Presentation

**DOI:** 10.1155/2016/2568031

**Published:** 2016-09-27

**Authors:** Nghi C. Nguyen, Muhammad Shah, Leonard J. Appleman, Rahul Parikh, James M. Mountz

**Affiliations:** ^1^Division of Nuclear Medicine, Department of Radiology, University of Pittsburgh, Pittsburgh, PA, USA; ^2^Division of Hematology/Oncology, Department of Medicine, University of Pittsburgh, Pittsburgh, PA, USA

## Abstract

*Background and Purpose*. Radium-223 dichloride (Xofigo®, Bayer HealthCare Pharmaceuticals Inc.) is the first *α*-particle emitter therapeutic agent approved by the FDA, with benefits in overall survival and delay in symptomatic skeletal event for patients with metastatic castrate-resistant prostate cancer (CRPC). Recent post hoc analyses of the phase III ALSYMPCA trial support the previously established safety profile as well as therapeutic effect and clinical outcome of Radium-223. Currently, Radium-223 is approved as a single agent therapy for metastatic CRPC. Clinical trials are currently investigating Radium-223 in additional clinical settings such as earlier asymptomatic disease and in combination with other agents including hormonal therapeutic agents and immunotherapeutic as well as chemotherapeutic agents. Trials are also ongoing in patients with other primary cancers such as breast cancer, thyroid cancer, and renal cancer metastatic to bone. In this article, the physics and radiobiology, as well as a literature update on the use of Radium-223, are provided along with case presentations, aiming at a better appreciation of research data as well as the assimilation of research data into clinical practice.

## 1. Introduction

Prostate cancer is the most common cancer in men in the United States and the third leading cause of cancer death among men after lung cancer and colon cancer. Approximately 14.0% of men will be diagnosed with prostate cancer at some point during their lifetime according to the 2010–2012 data. In 2013, there were an estimated 2,850,139 men living with prostate cancer in the United States. Based on data from 2009–2013, the number of new cases of prostate cancer was 129.4 per 100,000 men per year, and the number of deaths was 20.7 per 100,000 men per year [1]. Up to 85% of prostate cancer patients present with localized disease, but nearly 40% ultimately develop metastatic disease. More than 90% of patients with advanced prostate cancer have bone metastases [2, 3]. Patients with metastatic prostate cancer are initially treated with medical or surgical androgen deprivation. However, progression in spite of castrate levels of testosterone inevitably develops, which has been termed castration-resistant prostate cancer (CRPC). The prognosis of CRPC is poor with shorter survival compared with those with castrate-sensitive disease. Almost half of CRPC patients develop significant bone pain, skeletal-related complications, or both, with complications including pathologic fractures, spinal compression, malignant hypercalcemia, and bone marrow suppression [4]. Bone metastases reduce the quality of life and warrant an adequate management to avoid functional impairments. Traditional treatment strategies include analgesics, external beam radiation therapy (EBRT), and bone acting agents such as bisphosphonates or the receptor activator of nuclear factor kappa-B ligand (RANKL) monoclonal antibody denosumab. Docetaxel was the first chemotherapeutic agent capable of improving overall survival; however, it is associated with significant hematologic and nonhematologic toxicity [5].

Unlike systemic chemotherapy, bone-seeking radiopharmaceuticals allow targeted radiation of the bone. Among bone-seeking radiopharmaceuticals, beta- (*β*-) emitting radionuclides such as Strontium-89 and Samarium-153 EDTMP have been used successfully for bone pain therapy in CRPC. In spite of their palliative benefit, these agents have not been found to have a positive impact on survival [6, 7]. A new bone-seeking radiopharmaceutical based on the alpha- (*α*-) particle emitter Radium-223 dichloride (Xofigo, Bayer HealthCare Pharmaceuticals Inc.) has shown promise not only in bone pain relief but also in improving survival. Radium-223 received FDA approval in May 2013 following the phase III ALSYMPCA trial (ALpharadin in SYMptomatic Prostate CAncer), which was an international, multicenter, randomized, double-blind, placebo-controlled study of patients with symptomatic progressive CRPC who had previously received or were unfit for docetaxel chemotherapy [3]. In this review article, we summarize the physics and radiobiology of Radium-223 and provide a literature update on its use along with case presentations to facilitate at a better appreciation of research data as well as assimilation of research data into clinical practice.

## 2. Radium-223 Physics and Radiobiology

An *α*-particle is a positively charged ionized helium nucleus. The *α*-particle Radium-223 is an elemental chemical in the alkaline metal family of the periodic table and was the first in its class to obtain FDA approval for the management of bone pain in patients with CRPC in May 2013. It has a half-life of 11.4 days. Although present naturally in small amounts, Radium-223 is manufactured for medicinal purposes by neutron bombardment of Radium-226, which has a half-life of 1,590 years. Radium-223 decays to a stable isotope of lead (Pb-207) after emitting four *α*-particles with half-lives ranging from milliseconds to 36 minutes. The target of the intravenous (IV) Radium-223 injection is hydroxyapatite (Ca_5_[PO_4_]_3_OH), which is the primary component of the inorganic bone matrix. Radium-223 will localize to areas of osteoblastic metastases that contain hydroxyapatite [8].

Compared to other subatomic particles emitted from decaying radionuclides, *α*-particles are heavy, highly ionizing and capable of depositing a large amount of energy over a short range in tissue (50–100 *μ*m). The energy deposited is 1500 times more per unit path length compared to *β*-particles, rendering *α*-radiation more cytotoxic. The malignant cells can be targeted due to the high linear energy transfer (100 keV/*μ*m) of *α*-radiation, thus sparing the normal structures. The favorable characteristics of *α*-emitters including dose deposition, specificity, and low toxicity render it more suitable for cancer therapy compared with *β*-emitters [6, 7].

As an *α*-particle emitting radiopharmaceutical, Radium-223 faces some challenges regarding dosimetry [8, 9]. The traditional dosimetry guidelines presented by the Medical Internal Radiation Dose (MIRD) Committee are optimal for situations in which single dosimetry parameter values (e.g., mean absorbed dose to a target volume) are expected to reflect appropriately biological effect [9]. In practical clinical settings, however, the short range of *α*-particles relative to the human organ dimensions and associated critical structures as well as target volume leads to a highly nonuniform irradiation of the target volume. Currently, the radiobiology of *α*-particle emitters in humans is not clearly understood. It should, however, be noted that the relative biological effectiveness has been consistently found higher than unity. The likelihood of cellular death rendered by *α*-particles is greater than for *β*-emitters or photons for an equal amount of energy deposited in a cell [8, 9]. Alpha emission induces predominantly nonrepairable DNA double-strand breaks in cells which account for the antitumor characteristics of Radium-223 [10].

## 3. Pharmacokinetics

After IV injection, Radium-223 is rapidly cleared from the blood and is incorporated primarily into bone and bone metastases or is excreted into the intestine [11, 12]. Fifteen minutes after injection, about 20% of the injected activity remains in the blood. At 4 hours, about 4% of the injected activity remains in the blood, decreasing to less than 1% at 24 hours after the injection, while the activity in bone at 4 hours is between 44 and 77%. Fecal excretion is the major route of elimination from the body. About 5% is excreted in the urine. Radium-223 is neither metabolized by the liver nor eliminated via the bile. At seven days after injection, approximately 76% of administered activity has been cleared from the body. It should be noted however that the variability in intestinal transit will affect the rate of elimination of Radium-223 from the gastrointestinal tract.

## 4. Safety

The safety profile of Radium-223 is based on data from more than 1000 patients treated in the phase II and III studies [3, 13, 14]. Radium-223 therapy is considered safe and well tolerated, based upon updated data from a 3-year follow-up [15]. The most frequently observed adverse reactions (≥10%) were diarrhea, nausea, vomiting, and peripheral edema. The most common hematologic abnormalities (≥10%) related to Radium-223 administration were anemia, lymphocytopenia, leukopenia, thrombocytopenia, and neutropenia. The most common hematologic abnormalities resulting in the discontinuation for Radium-223 were anemia (2%) and thrombocytopenia (2%). Among the 13 patients who experienced bone marrow failure, 54% required blood transfusions. There were two deaths due to bone marrow failure [16]. In their first clinical experience, Nilsson et al. reported mild myelosuppression occurring 2 to 4 weeks after a single dose administration of Radium-223 in five different doses (46, 93, 163, 213, and 250 kBq/kg), which was reversed by 6 to 8 weeks in most patients [11]. In a recent retrospective study as part of an early access trial, 25 patients with a median of 5 monthly doses of 50 kBq/kg Radium-223 have been evaluated so far. The nadir of neutrophil counts and platelet counts was around 90 days (3 months) for both patients receiving Radium-223 alone (*n* = 11) and in combination with enzalutamide (*n* = 8) or abiraterone (*n* = 6) [17]. No statistically significant difference was observed in the mean change of blood counts between patients receiving Radium-223 alone and those receiving concurrent therapy with enzalutamide or abiraterone. However, there was a trend in thrombocytopenia in the concurrent group that did not reach statistical significance. The concurrent administration of Radium-223 with enzalutamide or abiraterone appears well tolerated with similar toxicities to the standard Radium-223 therapy [17].

## 5. Clinical Trials

A total of 922 patients participated in the phase III ALSYMPCA trial [18]. Patients were randomized in 2 : 1 fashion to six intravenous injections of Radium-223 administered every 4 weeks (50 kBq/kg body weight) or placebo in conjunction with best standard of care, which included therapies such as bicalutamide, dexamethasone, and flutamide, as well as bisphosphonates and EBRT. Patients with visceral disease or lymphadenopathy >3 cm in short axis diameter were excluded from participating.

The interim analysis demonstrated marked clinical benefit from Radium-223 therapy, leading to early completion of the trial. There was a statistically significant improvement in overall survival (OS) with Radium-223 treatment versus placebo (14.0 months versus 11.2 months; HR = 0.70; 95% CI 0.55–0.88; *p* = .002). Additional analyses were performed after the initial interim analysis and before crossover, which reported a median OS of 14.9 months for the Radium-223 group versus 11.3 months for the placebo group (HR = 0.70; 95% CI 0.58–0.83; *p* < .001). Importantly, both subgroups previously treated with docetaxel (HR = 0.71; 95% CI, 0.56–0.89) and not previously treated with docetaxel (HR = 0.74; 95% CI, 0.56–0.99) showed improved OS after Radium-223 administration.

Secondary endpoints including symptomatic skeletal events (SSE), time to alkaline phosphatase (ALP) increase, and time to prostate-specific antigen (PSA) progression were evaluated. Median time to first SSE was 15.6 months with Radium-223 compared with 9.8 months with placebo (HR = 0.66; 95% CI 0.52–0.83; *p* < .001). A significant number of patients treated with Radium-223 experienced 30% or greater reduction in total ALP level compared with placebo (47% versus 3%), and the time to increase in ALP level was prolonged (7.4 months versus 3.8 months). Although statistically significant, the changes for PSA level were less pronounced compared to ALP level, as 16% of patients receiving Radium-223 and 6% of placebo subjects showed 30% or greater reduction in PSA level at 12 weeks, and the time to increase in PSA level was minimal compared with placebo (3.6 months versus 3.4 months). At this time, ALP has emerged as the leading biomarker for Radium-223 treatment response. Similarly, ALP has previously been linked to survival in studies with agents such as docetaxel [19], but the role of ALP endpoint as a predictor for survival will need further exploration and validation.

Detailed analyses of SSEs were published separately in 2014 [20]. Radium-223 significantly prolonged the time to first SSE and reduced the risk of EBRT for bone pain and spinal cord compression, which represented the two most common types of SSEs. The risks of symptomatic pathologic bone fracture, as well as the need for metastasis-related orthopedic intervention, were however not statistically significantly different between the two groups. The effects of Radium-223 on SSEs were consistent regardless of prior docetaxel therapy, baseline ALP level, or use of bisphosphonates. Multivariate analyses indicated that bisphosphonate therapy reduced the SSE rate independent of Radium-223.

## 6. Clinical Indications

Radium-223 is indicated for the treatment of adult men with castration-resistant prostate cancer with symptomatic bone metastases and no known visceral metastases. Radium-223 should be administered only by authorized users in designated clinical settings. The IV injection is administered over one minute. The dose regimen is 50 kBq Radium-223 per kg body weight, given at 4-week intervals for a total of 6 injections. A dose adjustment is not necessary in elderly patients as no overall differences in safety or efficacy were seen between elderly (aged ≥ 65 years) and younger patients (aged < 65 years). No dose adjustment is deemed necessary in patients with renal or hepatic impairment. There are no restrictions regarding contact with other people after the Radium-223 administration. Patients may return home after the injection.

Bone marrow suppression is the main concern for Radium-223 therapy. Thus, a hematological evaluation must be performed at baseline and before each dose of Radium-223. Before Radium-223 initiation, the absolute neutrophil count (ANC) should be ≥1.5 × 10^9^/L, the platelet count ≥100 × 10^9^/L, and hemoglobin ≥10.0 g/dL. Before each subsequent Radium-223 administration, the ANC should be ≥1.0 × 10^9^/L and the platelet count ≥50 × 10^9^/L. If the ANC or platelet count does not recover within six weeks of the last Radium-223 administration, despite proper standard clinical management, further treatment with Radium-223 should only be considered after a careful assessment of risks and benefits.

## 7. Case Series

We have treated 45 clinical patients to date with Radium-223 for metastatic CRPC at the Division of Nuclear Medicine, Department of Radiology, University of Pittsburgh Medical Center. We chose these three clinical cases because the patients have had the longest follow-up and shown different clinical outcomes after Radium-223 therapy. Thus, the case selection was nonrandom.

### 7.1. Case #1

**Figure 1:**
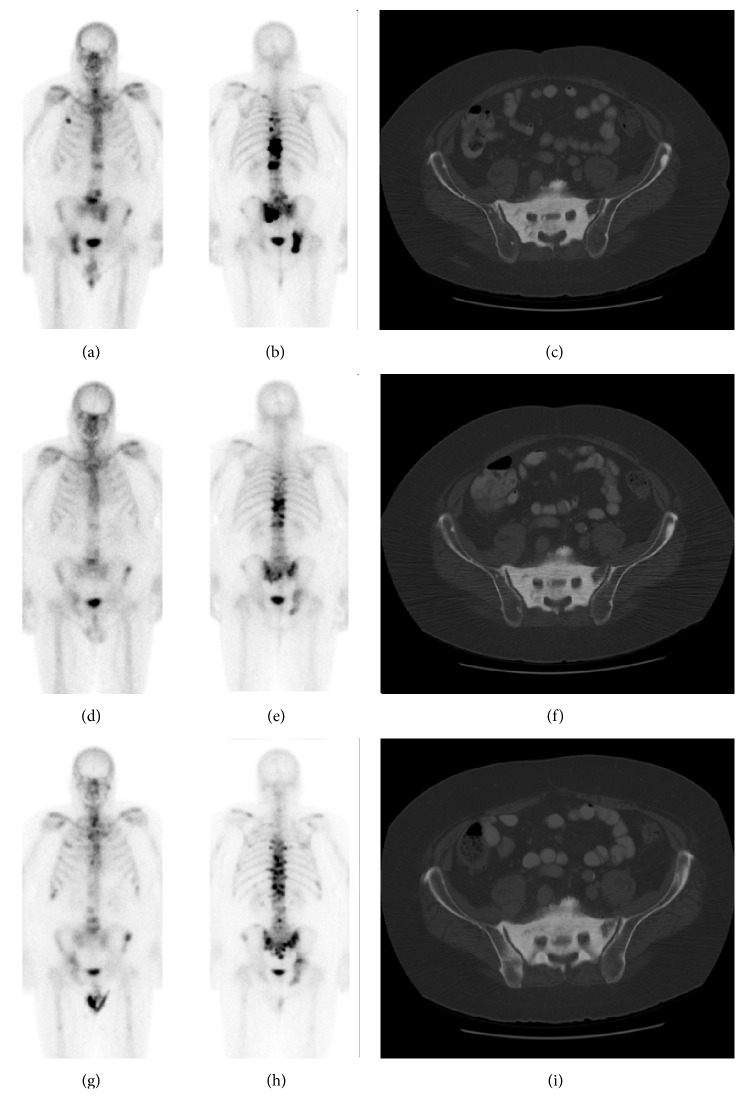
Case #1 of a 71-year-old man. Anterior and posterior bone scan, and CT at baseline (a, b, c), within 3 months after completion of Radium-223 (d, e, f), and 6–12 months after Radium-223 (g, h, i). Imaging findings are summarized in Table 1.

This 71-year-old man was diagnosed with Gleason score 7 prostate adenocarcinoma fifteen years before Radium-223 treatment and underwent radical prostatectomy and salvage radiation at that time. PSA recurrence was diagnosed four years after initial diagnosis and bone metastasis one year before Radium-223. Prior drug therapies included leuprolide acetate (Lupron) and then the addition of bicalutamide (Casodex), sipuleucel-T (Provenge), ketoconazole with hydrocortisone, and enzalutamide (Xtandi) and denosumab (Xgeva). The indication for Radium-223 therapy was progressive bone metastasis with lower back pain. He had back pain radiating to his hips which did not change following Radium-223 therapy and was thought to be secondary to spinal stenosis and not due to bone metastasis. The patient also received denosumab and leuprolide acetate during the Radium-223 treatments. Subsequently, the patient completed therapy with 8 months of oral metronomic cyclophosphamide, following which he decided to stop any additional therapy for his castrate-resistant prostate cancer. The patient is alive 25 months after Radium-223 therapy and shows evidence of progressive bone metastasis (Figure 1, Table 1). There is, however, no evidence of visceral metastasis or lymphadenopathy. Pain level has remained constant.

### 7.2. Case #2

**Figure 2:**
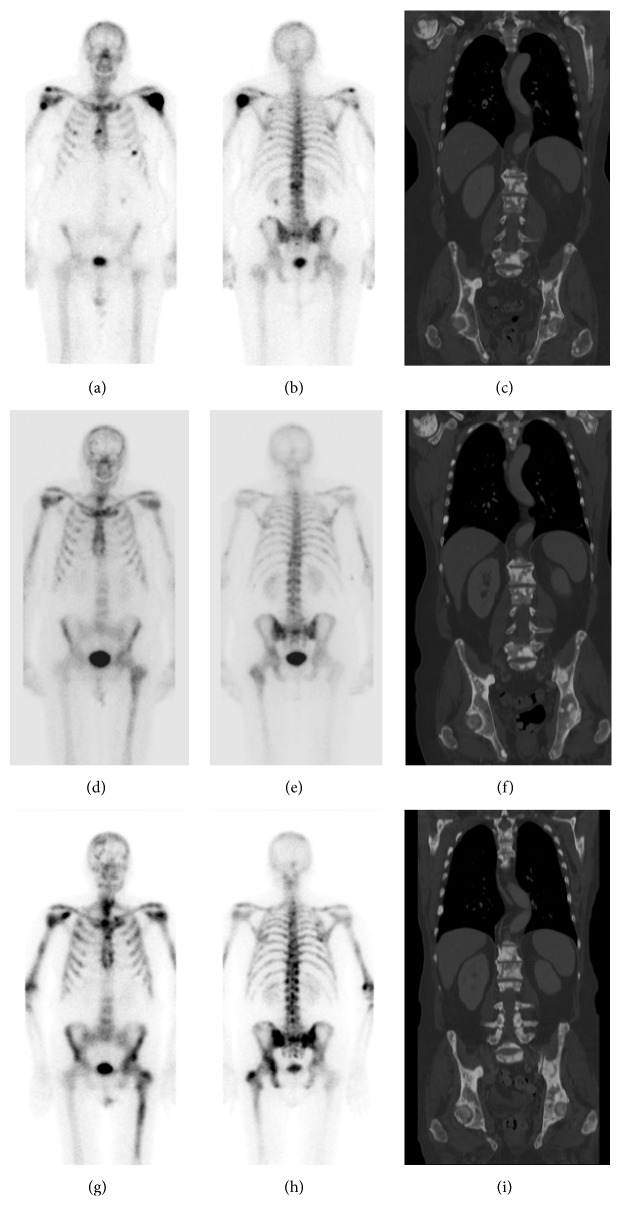
Case #2 of a 65-year-old man. Anterior and posterior bone scan, and CT at baseline (a, b, c), within 3 months after completion of Radium-223 (d, e, f), and 6–12 months after Radium-223 (g, h, i). Imaging findings are summarized in Table 2.

This 65-year-old man had been diagnosed with Gleason score 8 prostate adenocarcinoma nine years before Radium-223 treatment and underwent radical prostatectomy and salvage radiation at that time. PSA recurred five years later which was treated with single agent bicalutamide, followed by leuprolide acetate (Lupron), bicalutamide (Casodex), sipuleucel-T (Provenge), and docetaxel (Taxotere) 18 months before Radium-223, as well as abiraterone (Zytiga). The indication for Radium-223 therapy was progressive bone metastasis with pain to the left shoulder, lower back, and right hip. The patient did experience pain relief from Radium-223 therapy. He received red blood cell transfusion because his Hb decreased to 7.3, and he complained of severe fatigue two months after Radium-223. He also developed pancytopenia which was attributed to Radium-223 therapy and potential bone marrow involvement by disease (bone marrow biopsy was declined). Peripheral smear showed normocytic normochromic anemia with a few tear drops and some polychromasia. There was no evidence of dysplasia in the granulocyte cell lines. After Radium-223 therapy, he received denosumab (Xgeva) and leuprolide acetate (Lupron) as well as enzalutamide (Xtandi). The patient died 19 months after Radium-223 therapy with progressive bone metastasis as well as visceral metastases and lymphadenopathy (Figure 2, Table 2).

### 7.3. Case #3

**Figure 3:**
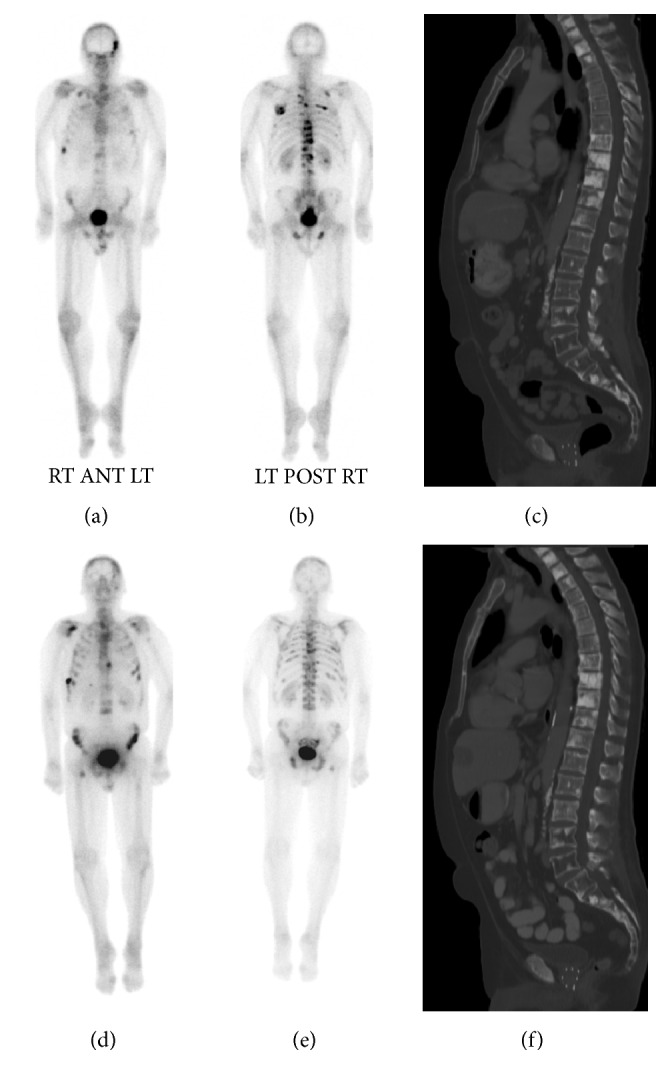
Case #3 of a 67-year-old man. Anterior and posterior bone scan, and CT at baseline (a, b, c) and within 3 months after completion of Radium-223 (d, e, f). Imaging findings are summarized in Table 3.

This 67-year-old man was diagnosed with Gleason score 7 prostate adenocarcinoma seven years before Radium-223 treatment and was treated with EBRT plus prostate seed. His PSA recurred one year later, for which he received bicalutamide (Casodex), leuprolide acetate (Lupron), ketoconazole, and corticosteroid. He developed bone metastasis to the lumbosacral spine requiring EBRT for two years and received 81 mCi radioactive Samarium-153 lexidronam (Quadramet®) one year before Radium-223. Subsequently, the patient participated in phase II clinical trial with an antibody-drug conjugate but the treatment was stopped shortly before Radium-223 therapy due to severe peripheral neuropathy of hands and feet. The indication for Radium-223 treatment was progressive bone metastasis with mild low back pain. There were suspicious small liver metastases at the time of Radium-223 initiation. His low back pain disappeared during the first two Radium-223 infusions but recurred afterward, and additional pain around the shoulder regions had developed. He developed grade 2 anemia with Hb 8.5 mg/dL within three months of completion of Radium-223 and was found to have progressive disease involving the bone, liver, lymph nodes, lungs, and brain (Figure 3, Table 3). He was then treated with low-dose, daily cyclophosphamide (Cytoxan) and degarelix (Firmagon) but died five months after Radium-223 therapy.

## 8. Discussion

Radium-223 therapy has been shown to have a favorable therapeutic effect, good tolerance, and low toxicity. Compared with *β*-emitters, the *α*-emitter Radium-223 appears to have the advantage of sparing much of the bone marrow from irradiation given its short-range emissions [21, 22]. Radium-223 myelotoxicity is infrequent [16]. No cases of myelodysplastic syndrome, acute myelogenous leukemia, or aplastic anemia have been found in 2-year and 3-year follow-up [18, 23]. The cause of the grade 2 anemia observed in Case #3 was multifactorial. The patient was found to have extensive progressive bone disease as well as worsening of visceral metastasis and lymphadenopathy soon after Radium-223 therapy and was treated with low-dose daily cyclophosphamide before his death five months after Radium-223 therapy. His bone marrow toxicity was likely a combination of prior Samarium-153 lexidronam therapy, extensive bone marrow involvement, and Radium-223 side effect. Grade 3-4 pancytopenia is rare (1%) [3, 16]. However, Case #2 did develop grade 3 pancytopenia (most notably Hb < 8.0; platelets < 50,000), which was likely attributed to Radium-223 therapy. In the phase III ALSYMPCA trial, grade 3-4 thrombocytopenia occurred in 1% of patients not previously treated with docetaxel and in 4% of patients who had received prior docetaxel. Thus, patients who received prior docetaxel may have a greater risk of developing thrombocytopenia with Radium-223. Grade 3-4 neutropenia has occurred in 1% of patients not previously treated with docetaxel and in 3% of patients who have received prior docetaxel. The concurrent use of Radium-223 and docetaxel is not advised in a clinical setting, but the safety and efficacy of combination therapies are being evaluated in clinical trials. A phase 1/2a clinical trial presented by Morris et al. at the 2015 ASCO meeting showed that Radium-223 with docetaxel was particularly effective at normalizing bone ALP level compared with docetaxel alone [24].

Recent post hoc analyses of the ALSYMPCA data demonstrated that Radium-223 treatment was associated with greater pain relief compared with placebo at 16 weeks and 24 weeks after treatment (odds ratio 2.58; 95% CI 1.18–5.62; *p* = .018) [23]. Some patients however may not experience much pain relief, which we also observe in our clinical practice. Given the favorable therapeutic profiles with increased OS and delay in SSEs, a lack of pain response should not discourage the patient to continue with Radium-223 treatment. Instead, the pain medication should be adjusted as needed to ensure that the patient can complete all six Radium-223 injections. Another post hoc analysis also showed that the hematologic safety profile of concomitant administration of Radium-223 and EBRT was similar to Radium-223 therapy without EBRT [25]. However, further studies are necessary to confirm this observation.

In Cases #1 and #2, the striking reduction in ALP was associated with a significant decrease in radiotracer uptake at bone scanning. ALP then increased within 6–12 months after Radium-223 but the increase was still below the baseline value, which correlated well with a relatively mild increase in radiotracer uptake. In a phase II trial, the median change in bone ALP was −65.6% in the Radium-223 group compared with −9.3% in the placebo group 4 weeks after completion of therapy [26]. A report by Nome et al. showed that ALP was decreased in nine of 14 patients three months after treatment initiation. Four weeks after the final Radium-223 injection, six subjects were found to have ALP values equal to or 30% below the baseline [27]. They further demonstrated that, in 10 of the 12 patients with available bone scanning, a reduction in radiotracer uptake was noticed in lesions with high pretreatment radiotracer uptake. However, new metastatic radiotracer foci developed in 11 of the 12 patients, which was also observed in our case series at 6–12-month follow-up. Changes in ALP, as a secretory product of osteoblasts and changes in uptake intensity on bone scan serve as valuable biomarkers for treatment monitoring of bone metastasis [3, 26, 27]. However, these suppressive effects are mostly temporary, resulting in the development of new sites of bone metastases within 6–12 months of Radium-223. In addition, tumor recurrence seen as interval increased radiotracer uptake at initial metastatic sites may also be observed [27]. The ALSYMPCA trial also suggested that patients with fewer bone metastases on bone scanning would benefit less from Radium-223 monotherapy [3]. Further studies are required to determine how much Radium-223 can affect specific osteoblast-derived factors that play an essential role in homing, dormancy, and colonization as well as proliferation of bone metastasis. An observational, prospective, single-arm cohort trial is currently under way to assess OS, SSE-free survival, and quality of life in chemonaïve metastatic CRPC patients receiving Radium-223 under real-life conditions in Germany (NCT02450812).

In regard to PSA levels, all patients in this case series showed rising PSA during Radium-223 therapy. Particularly, the rising PSA in Case #1 was not associated with a significant burden of visceral metastasis or lymphadenopathy, and it was in contrast to the decline of ALP as well as decreased uptake on bone scanning. This observation is, however, consistent with literature reports indicating that a treatment response of bone metastasis may not be associated with a decline in PSA [3, 27]. In the ALSYMPCA trial, only 16% of patients had a 30% or greater reduction in the PSA level at 12 weeks [3].

Case #1 has no evidence of visceral metastasis or lymphadenopathy and is still alive 25 months after Radium-223. Case #2 was found to have a stable 1.1 cm external iliac lymphadenopathy at Radium-223 initiation, which was however exacerbated during Radium-223; this patient died 19 months after Radium-223 therapy. Case #3 was found to have several small suspicious liver metastases at Radium-223 initiation; this patient died five months after completion of Radium-223. These findings are consistent with the notion that Radium-223 is most appropriate for patients with primary bone metastases. Monotherapy with Radium-223 may be considered a poor therapeutic option in patients with visceral metastasis or lymphadenopathy because of the minimal gain in overall survival [3]. A careful consideration of pain symptoms and tumor burden, of both visceral metastasis and lymphadenopathy, is necessary for an optimal patient selection for Radium-223 treatment

Several hormonal therapeutic agents were approved in the last 7 years including abiraterone, enzalutamide, and bicalutamide; immunotherapeutic agents such as sipuleucel-T; RANK ligand inhibitors such as denosumab; and the chemotherapeutic agent cabazitaxel. The efficacy and safety of Radium-223 in combination with some of these newer drugs are being evaluated in clinical trials [23]. Preliminary results of a phase IIIb early access study involving 696 patients showed that the concomitant treatment of Radium-223 with abiraterone or denosumab appeared to increase OS compared with Radium-223 alone [28]. Particularly, statistically significantly longer OS was observed in patients with low Eastern Cooperative Oncology Group (ECOG) scores 0-1, no bone pain, and low ALP (less than 220 U/L). However, grade 3 and 4 adverse events were reported in 38% of patients, and Radium-223 therapy was discontinued in 21% of patients due to adverse events [28]. Preliminary data of two studies in an expanded access setting showed that the safety profiles of Radium-223 with or without concurrent abiraterone or enzalutamide were similar [17, 29]. Also, there may be additive or synergistic effect of Radium-223 and these hormonal drugs [29]. It is expected that more therapeutic options for management of metastatic CRPC will be available in the coming years.

The evaluation of bone metastases in CRPC remains a challenge in clinical practice because they are difficult to measure. The updated Response Evaluation Criteria in Solid Tumors (RECIST) 1.1 criteria published in 2009 are commonly used to measure treatment response in oncology; however, they mainly address soft tissue lesions and lack an adequate evaluation of bone lesions [30]. Metastatic bone lesions are considered target lesions only if they demonstrate associated soft tissue component measuring ≥10 mm, which would exclude the majority of bone metastasis from prostate cancer. In an attempt to address the difficulty of evaluating bone metastasis, the MD Anderson Cancer Center criteria introduced in 2004 provided specific criteria to assess bone metastasis based on X-ray, bone scan, CT, and MRI. In the recent St. Gallen Advanced Prostate Cancer Consensus Conference 2015, the panel recommends regular imaging with CT scan (of the chest, abdomen, and pelvis) and bone scan for monitoring of therapy [31]. For patients undergoing Radium-223, the majority of the panel recommends CT scans every 2–4 months or every 6 months; 30% of the panel recommends CT only if clinically indicated. No specific comments on the use of bone scan were made; however, the panel acknowledged that the clinical value and image interpretation are not well characterized for treatment monitoring. For both bone scan and CT scan, the potential pitfall of flare phenomenon, a spurious increase in radionuclide uptake and bone density due to reparative mechanism associated with treatment response, needs to be considered to avoid false-positive image interpretation [32–34]. In addition, the diagnostic accuracy of bone scan and CT scan lags behind that of more advanced imaging techniques (e.g., whole-body MRI and F-18 sodium fluoride (F-18 NaF) PET/CT) and thus may result in underestimation of lesions as well as suboptimal monitoring of bone disease [35–41]. Advanced imaging techniques are however associated with higher medical cost and not readily available in many imaging centers; and F-18 NaF PET is currently restricted to coverage with evidence development by the US Centers for Medicare and Medicaid Services (CMS). Moreover, the incremental values of advanced imaging biomarkers compared with conventional CT scan and bone scan in treatment monitoring and clinical outcome have not been evaluated in randomized prospective trials. Yet, the latest National Comprehensive Cancer Network (NCCN) guideline update on prostate cancer, Version 3.2016, does include advanced imaging biomarkers [42]. For MRI, diffusion-weighted imaging, spectroscopy, and dynamic-contrast enhanced imaging have been suggested. PET/CT biomarkers may include F-18 NaF PET/CT, FDG PET/CT, and C-11 choline PET/CT.

The potential role of Radium-223 in the treatment of osteoblastic bone metastasis from other primaries such as breast cancer, thyroid cancer, and renal cancer is currently under investigation [43, 44]. In a phase II trial, patients with predominant HER2-negative, hormone receptor-positive bone metastasis will receive either Radium-223 or placebo, with both arms receiving background hormonal therapy (NCT02258464). Another phase II trial is evaluating the metabolic response of Radium-223 in the treatment of radioiodine refractory bone metastasis from thyroid cancer (NCT02390934). In a phase I trial, the combination of Radium-223 and VEGF-targeted therapy in bone metastasis from renal cell cancer is being evaluated (NCT02406521).

## 9. Conclusion

Radium-223 is the first *α*-particle emitter therapeutic agent approved by the FDA that has shown benefits in overall survival and delay in symptomatic skeletal event in the phase III ALSYMPCA trial. Recent post hoc analyses support the previously established safety profiles as well as therapeutic effect and clinical outcome of Radium-223. The role of Radium-223 to manage micro bone metastases in early metastatic CRPC is yet to be determined. Ongoing clinical trials with Radium-223, particularly in combination with other agents, will examine ways to further improve patient outcome in advanced disease. Trials are also ongoing in patients with other primary cancers such as breast cancer, thyroid cancer, and renal cancer metastatic to bone.

## Figures and Tables

**Table 1:** Case #1: summary of pertinent laboratory results and imaging findings.

Lab work/imaging (normal values)	Baseline (within 3 mo before Radium-223)	Interim (between injections #3 and #4)	Completion (within 3 mo after Radium-223)	Follow-up (6–12 mo after Radium-223)
Hb (12.9–16.9/dL)	14.6	13.6	12.8	13.2
Platelets (156–369 × 10^9^/L)	272	256	214	267
Absolute neutrophils (2.240–7.680 × 10^9^/L)	3.000	2.100	3.200	4.100
PSA (ng/mL)	133	232	385	1283
Alkaline phosphatase (38–126 IU/L)	123	51	78	234
Whole-body bone scan	Widespread bone metastases with the most intense lesions located along the T-spine, L-spine, and pelvis		Previous bone metastases demonstrate interval decrease in uptake. No evidence of new bone metastasis	Some of previous skeletal metastases show interval increase in uptake; others show stable uptake. A few new tracer foci in the L-spine and pelvis are suspicious for metastases
Diagnostic CT-chest, abdomen, and pelvis	Widespread bone metastases consistent with bone scan. No evidence of soft tissue metastasis		No significant change in bone metastasis compared to baseline study. No evidence of visceral metastasis or lymphadenopathy	A few areas of diffuse osteosclerosis have developed, for example, right posterior iliac bone consistent with bone scan. Otherwise no significant change in bone metastases

**Table 2:** Case #2: summary of pertinent laboratory results and imaging findings.

Lab work/imaging (normal values)	Baseline (within 3 mo before Radium-223)	Interim (between injections #3 and #4)	Completion (within 3 mo after Radium-223)	Follow-up (6–12 mo after Radium-223)
Hb (12.9–16.9/dL)	11.2	11.0	8.4	9.6
Platelets (156–369 × 10^9^/L)	209	141	119	37
Absolute neutrophils (2.240–7.680 × 10^9^/L)	4.400	1.500	1.300	2.100
PSA (ng/mL)	33	168	310	396
Alkaline phosphatase (38–126 IU/L)	110	79	94	201
Whole-body bone scan	Widespread bone metastases including bilateral proximal humerus, right parasagittal skull, sternum, ribs, spine, and pelvis		Previous bone metastases show interval decrease in uptake. New foci of mildly increased uptake are present in the right skull, ribs, right acromion and left femur	Significant increase in bone uptake, less pronounced in previously treated lesions and more pronounced in lesions developing after Xofigo
Diagnostic CT-chest, abdomen, and pelvis	Widespread bone metastases consistent with bone scan. No evidence of local recurrence in the prostatectomy bed. Stable 1.1 cm right external iliac chain node		No significant change in bone metastases. There is new lymphadenopathy including the axillae, mediastinum, retroperitoneum, and pelvis	Progression of widespread bone metastases consistent with bone scan as well as extensive lymphadenopathy

**Table 3:** Case #3: summary of pertinent laboratory results and imaging findings.

Lab work/imaging (normal values)	Baseline (within 3 mo before Radium-223)	Interim (between injections #3 and #4)	Completion (within 3 mo after Radium-223)
Hb (12.9–16.9/dL)	11.5	10.9	8.5
Platelets (156–369 × 10^9^/L)	231	273	157
Absolute neutrophils (2.240–7.680 × 10^9^/L)	4.3	5.3	5.5
PSA (ng/mL)	33	119	597
Alkaline phosphatase (38–126 IU/L)	56	69	246
Whole-body bone scan	Widespread bone metastases with the most intense lesions located along the T-spine, left skull, and ribs		Previous bone metastases show interval decrease in uptake. There are new bone metastases at other sites (upper T-spine, L-spine, pelvis, and ribs)
Diagnostic CT-chest, abdomen, and pelvis	Widespread bone metastases consistent with bone scan. Several small liver lesions suspicious for metastases. No evidence of lymphadenopathy		Stable osteoblastic metastases. New areas of mild osteosclerosis in T-spine and L- spine consistent with bone scan findings. Also, worsening of liver metastases and suspicious new pulmonary metastases

## References

[1] Howlader N., Noone A. M., Krapcho M. *SEER Cancer Statistics Review, 1975–2013*.

[2] Roodman G. D. (2004). Mechanisms of bone metastasis. *The New England Journal of Medicine*.

[3] Parker C., Nilsson S., Heinrich D. (2013). Alpha emitter radium-223 and survival in metastatic prostate cancer. *The New England Journal of Medicine*.

[4] Lien L. M. E., Tvedt B., Heinrich D. (2015). Treatment of castration-resistant prostate cancer and bone metastases with radium-223 dichloride. *International Journal of Urological Nursing*.

[5] Petrylak D. P., Tangen C. M., Hussain M. H. A. (2004). Docetaxel and estramustine compared with mitoxantrone and prednisone for advanced refractory prostate cancer. *The New England Journal of Medicine*.

[6] Buchali K., Correns H.-J., Schuerer M., Schnorr D., Lips H., Sydow K. (1988). Results of a double blind study of 89-strontium therapy of skeletal metastases of prostatic carcinoma. *European Journal of Nuclear Medicine*.

[7] Sartor O. (2004). Overview of samarium sm 153 lexidronam in the treatment of painful metastatic bone disease. *Reviews in Urology*.

[8] Sartor O., Hoskin P., Bruland Ø. S. (2013). Targeted radio-nuclide therapy of skeletal metastases. *Cancer Treatment Reviews*.

[9] Sgouros G., Roeske J. C., McDevitt M. R. (2010). MIRD pamphlet No. 22 (Abridged): radiobiology and dosimetry of *α*-particle emitters for targeted radionuclide therapy. *Journal of Nuclear Medicine*.

[10] Bruland O. S., Nilsson S., Fisher D. R., Larsen R. H. (2006). High-linear energy transfer irradiation targeted to skeletal metastases by the *α*-emitter 223Ra: adjuvant or alternative to conventional modalities?. *Clinical Cancer Research*.

[11] Nilsson S., Larsen R. H., Fossa S. D. (2005). First clinical experience with *α*-emitting radium-223 in the treatment of skeletal metastases. *Clinical Cancer Research*.

[12] Carrasquillo J. A., O'Donoghue J. A., Pandit-Taskar N. (2013). Phase I pharmacokinetic and biodistribution study with escalating doses of ^223^Ra-dichloride in men with castration-resistant metastatic prostate cancer. *European Journal of Nuclear Medicine and Molecular Imaging*.

[13] Nilsson S., Franzén L., Parker C. (2013). Two-year survival follow-up of the randomized, double-blind, placebo-controlled phase II study of radium-223 chloride in patients with castration-resistant prostate cancer and bone metastases. *Clinical Genitourinary Cancer*.

[14] Nilsson S., Strang P., Aksnes A. K. (2012). A randomized, dose-response, multicenter phase II study of radium-223 chloride for the palliation of painful bone metastases in patients with castration-resistant prostate cancer. *European Journal of Cancer*.

[15] Parker C., Vogelzang N. J., Sartor O. (2015). 3-year safety follow-up of radium-223 dichloride (Ra-223) in patients (Pts) with castration-resistant prostate cancer (CRPC) and symptomatic bone metastases (Mets) from ALSYMPCA. *Journal of Clinical Oncology*.

[16] (2013). *Xofigo® (Radium Ra 223 Dichloride) Injection [Prescribing Information]*.

[17] Dan T. D., Eldredge-Hindy H. B., Hoffman-Censits J. (2015). Hematologic toxicity of concurrent administration of radium-223 and next-generation antiandrogen therapies. *American Journal of Clinical Oncology*.

[18] Parker C. C., Pascoe S., Chodacki A. (2013). A randomized, double-blind, dose-finding, multicenter, phase 2 study of radium chloride (Ra 223) in patients with bone metastases and castration-resistant prostate cancer. *European Urology*.

[19] Sonpavde G., Pond G. R., Berry W. R. (2012). Serum alkaline phosphatase changes predict survival independent of PSA changes in men with castration-resistant prostate cancer and bone metastasis receiving chemotherapy. *Urologic Oncology: Seminars and Original Investigations*.

[20] Sartor O., Coleman R., Nilsson S. (2014). Effect of radium-223 dichloride on symptomatic skeletal events in patients with castration-resistant prostate cancer and bone metastases: results from a phase 3, double-blind, randomised trial. *The Lancet Oncology*.

[21] Hobbs R. F., Song H., Watchman C. J. (2012). A bone marrow toxicity model for 223Ra alpha-emitter radiopharmaceutical therapy. *Physics in Medicine and Biology*.

[22] Lassmann M., Nosske D. (2013). Dosimetry of 223Ra-chloride: dose to normal organs and tissues. *European Journal of Nuclear Medicine and Molecular Imaging*.

[23] Nilsson S. (2016). Radionuclide therapies in prostate cancer: integrating radium-223 in the treatment of patients with metastatic castration-resistant prostate cancer. *Current Oncology Reports*.

[24] Morris M., Higano C. S., Scher H. I. (2015). Effects of radium-223 dichloride with docetaxel versus docetaxel on prostate specific antigen (PSA) and bone alkaline phosphatase (bALP) in patients (pts) with castration resistant prostate cancer (CRPC) and bone metastases (mets): a phase 1/2a clinical trial. *Journal of Clinical Oncology*.

[25] Finkelstein S. E., Michalski J. M., O'Sullivan J. M. (2015). External Beam Radiation Therapy (EBRT) use and safety with radium-223 dichloride (Ra-223) in patients with Castration-Resistant Prostate Cancer (CRPC) and symptomatic bone metastases from the ALSYMPCA trial. *Journal of Clinical Oncology*.

[26] Nilsson S., Franzén L., Parker C. (2007). Bone-targeted radium-223 in symptomatic, hormone-refractory prostate cancer: a randomised, multicentre, placebo-controlled phase II study. *The Lancet Oncology*.

[27] Nome R., Hernes E., Bogsrud T. V., Bjøro T., Fosså S. D. (2014). Changes in prostate-specific antigen, markers of bone metabolism and bone scans after treatment with radium-223. *Scandinavian Journal of Urology*.

[28] Saad F., Carles J., Gillessen S. (2015). Radium-223 in an international early access program (EAP): effects of concomitant medication on overall survival in metastatic castration resistant prostate cancer (mCRCP) patients. *Journal of Clinical Oncology*.

[29] Sartor A. O., Fernandez D. C., Morris M. J. (2015). Prior and concurrent use of abiraterone and enzalutamide with Ra-223 in an expanded access setting. *Journal of Clinical Oncology*.

[30] Eisenhauer E. A., Therasse P., Bogaerts J. (2009). New response evaluation criteria in solid tumours: revised RECIST guideline (version 1.1). *European Journal of Cancer*.

[31] Gillessen S., Omlin A., Attard G. (2015). Management of patients with advanced prostate cancer: recommendations of the St Gallen Advanced Prostate Cancer Consensus Conference (APCCC) 2015. *Annals of Oncology*.

[32] Ryan C. J., Shah S., Efstathiou E. (2011). Phase II study of abiraterone acetate in chemotherapy-naive metastatic castration-resistant prostate cancer displaying bone flare discordant with serologic response. *Clinical Cancer Research*.

[33] Messiou C., Cook G., Reid A. H. M. (2011). The CT flare response of metastatic bone disease in prostate cancer. *Acta Radiologica*.

[34] Morris M. J., Molina A., Small E. J. (2015). Radiographic progression-free survival as a response biomarker in metastatic castration-resistant prostate cancer: COU-AA-302 results. *Journal of Clinical Oncology*.

[35] Padhani A. R., Makris A., Gall P., Collins D. J., Tunariu N., de Bono J. S. (2014). Therapy monitoring of skeletal metastases with whole-body diffusion MRI. *Journal of Magnetic Resonance Imaging*.

[36] Lecouvet F. E., Larbi A., Pasoglou V. (2013). MRI for response assessment in metastatic bone disease. *European Radiology*.

[37] Lecouvet F. E., Talbot J. N., Messiou C., Bourguet P., Liu Y., de Souza N. M. (2014). Monitoring the response of bone metastases to treatment with Magnetic Resonance Imaging and nuclear medicine techniques: a review and position statement by the European Organisation for Research and Treatment of Cancer imaging group. *European Journal of Cancer*.

[38] Shen C.-T., Qiu Z.-L., Han T.-T., Luo Q.-Y. (2015). Performance of 18F-fluoride PET or PET/CT for the detection of bone metastases: a meta-analysis. *Clinical Nuclear Medicine*.

[39] Bortot D. C., Amorim B. J., Oki G. C. (2012). ^18^F-Fluoride PET/CT is highly effective for excluding bone metastases even in patients with equivocal bone scintigraphy. *European Journal of Nuclear Medicine and Molecular Imaging*.

[40] Chakraborty D., Bhattacharya A., Mete U. K., Mittal B. R. (2013). Comparison of ^18^F fluoride PET/CT and^ 99m^Tc-MDP bone scan in the detection of skeletal metastases in urinary bladder carcinoma. *Clinical Nuclear Medicine*.

[41] Iagaru A., Mittra E., Dick D. W., Gambhir S. S. (2012). Prospective evaluation of (99m)Tc MDP scintigraphy, (18)F NaF PET/CT, and (18)F FDG PET/CT for detection of skeletal metastases. *Molecular Imaging and Biology*.

[42] National Comprehensive Cancer Network (NCCN) (2016). *Prostate Cancer Updates*.

[43] Takalkar A., Adams S., Subbiah V. (2014). Radium-223 dichloride bone-targeted alpha particle therapy for hormone-refractory breast cancer metastatic to bone. *Experimental Hematology & Oncology*.

[44] Coleman R., Aksnes A.-K., Naume B. (2014). A phase IIa, nonrandomized study of radium-223 dichloride in advanced breast cancer patients with bone-dominant disease. *Breast Cancer Research and Treatment*.

